# The Effect of Medication and Deep Brain Stimulation on Posture in Parkinson's Disease

**DOI:** 10.3389/fneur.2019.01254

**Published:** 2019-12-03

**Authors:** Christian Schlenstedt, Olga Gavriliuc, Kathrin Boße, Robin Wolke, Oliver Granert, Günther Deuschl, Nils G. Margraf

**Affiliations:** ^1^Department of Neurology, UKSH, Christian-Albrechts-University, Kiel, Germany; ^2^Department of Neurology, State University of Medicine and Pharmacy “Nicolae Testemitanu”, Chisinau, Moldova

**Keywords:** Parkinson's disease, posture, angle measurement, deep brain stimulation, camptocormia, forward bending, Pisa syndrome, postural abnormalities

## Abstract

**Introduction:** Postural abnormalities are common in Parkinson's disease (PD) and increasing with disease progression. While many studies focus on balance and gait, postural alignment is only infrequently studied.

**Purpose:** The aim of the present study was to examine the immediate and long-term effects of medication and deep brain stimulation (DBS) in the subthalamic nucleus on postural alignment in PD.

**Materials and Methods:** PD patients (*n* = 192) in an advanced stage of disease were videotaped during a standardized l-dopa trial before and after DBS. The patients were tested with and without medication pre-surgical and retested post-surgical (6–24 months) in all treatment combinations of medication and DBS regarding the on and off conditions. The forward bending as total camptocormia (TCC) and upper camptocormia (UCC) angles and lateral bending as Pisa angle were assessed with the free downloadable NeuroPostureApp (http://www.neuroimaging.uni-kiel.de/NeuroPostureApp/). Three subgroups were defined according to normative values of healthy controls and according to clinical criteria: patients with normal posture, with stooped posture, and with postural disorders.

**Results:** A stooped posture was found in 82% of the patients with regard to the TCC angle and in 54% for the UCC angle. Sixty-two percent had an abnormal Pisa angle. Camptocormia was diagnosed in ~7% and a Pisa syndrome in 1% of the patients. Medication and DBS both significantly improved postural alignment in the entire cohort. Female and male patients benefit similarly by medication and stimulation. Subgroup analyses revealed that the effects were also significant for patients with stooped posture, and the effects were strongest for patients with camptocormia: they led to angles below the diagnostical criterion for camptocormia for 13 of 14 patients with TCC and 11 of 26 patients with UCC. DBS had an additional effect to medication over time for the Pisa angle.

**Conclusion:** Medication and DBS both improved postural alignment in PD patients, but effects were small for the entire cohort. Patients with camptocormia according to the TCC angle benefit strongest. The large differences of the treatment effects may indicate distinct pathological mechanisms for stooped posture and postural disorders. The TCC angle was shown to be sensitive to change. The UCC angle was less sensitive but may be a useful assessment tool for a subgroup.

## Introduction

With disease progression in Parkinson's disease (PD), postural disorders become more apparent and increase the risk of falls or injury and reduce the quality of life (1). The spectrum encompasses, in addition to the frequently encountered stooped posture with mildly bent hip and knee, the postural changes of camptocormia, a pathological non-fixed forward bending, and the dropped head and Pisa syndrome, the latter as marked lateral bending of the trunk (2). While treatment with deep brain stimulation (DBS) or with medication, such as l-dopa or dopamine agonists, is effective in improving several symptoms of the disease, controversy exists with regard to the axial symptoms (3). Curtze et al. have shown that some features of static and dynamic postural control improve under l-dopa, whereas others do not (4). With respect to static postural control, another study showed a medication-induced increase in the postural sway in the mediolateral direction and a decrease in postural sway by DBS (5). A study investigating the effect of l-dopa and DBS on reactive postural control did not demonstrate any improvement (6). While these studies focus on postural control mechanisms, such as postural reactions to perturbations or postural sway during quiet relaxed stance, there are fewer studies on the effect of medication or DBS on postural alignment. A recently published study investigated the effect of DBS on postural alignment and found improved posture by DBS (7). However, the immediate and long-term effects of l-dopa compared to DBS as well as the combined effect of both on postural alignment in the anteroposterior and mediolateral direction still remain to be identified.

An easily applicable method provided as a free Webapp was recently introduced to assess the pathological alterations of posture, especially of postural alignment, and to describe these disorders in an objective way, allowing one to investigate and test larger groups (8). In another paper (Schlenstedt et al., submitted), we have confirmed the reliability of the method and focused on a description of posture in healthy individuals and PD patients. Here, we used the same method to analyze different treatment effects on posture in a large cohort of patients with advanced PD.

This study had the following aims: First, we investigated the immediate effect of medication (Med) and DBS (Stim) on posture in patients with PD and in subgroups of PD patients with normal posture, impaired (stooped) posture, or clinically diagnosed camptocormia. Second, we studied whether the long-term treatment with DBS has a carryover effect on posture. We compared the medication Off-state before surgery with the Med-Off/Stim-Off condition at follow-up to determine whether the treatment with DBS impacted posture over time. Third, we investigated whether DBS had an effect additional to best medical treatment over time. The baseline condition Med-On was compared to follow-up Med-On/Stim-On. Fourth, we investigated whether the effect of medication or stimulation differed between male and female patients. Finally, predictors of the effect of DBS were identified, and clinical conclusions from these measurements were drawn.

## Materials and Methods

### Patients

The DBS video database of the Neurology Department at the University of Kiel, Germany was used for this study. Patients were included if they suffered from idiopathic PD according to the UK Brain Bank PD criteria (9), had received DBS of the subthalamic nucleus, and if medical records and a full set of a videotaped standardized l-dopa trials were available before electrode implantation and at follow-up for up to 2 years (Table 1). A total of 192 patients were included. Exclusion criteria were any other neurological disease, or any diseases or injuries, which could affect gait and posture.

**Table 1:** Patient characteristics (pre-surgery).

**Variable**	**Value**
Age (years)	59.2 (8.8)
Duration of PD (years)	13.3 (5.1)
Gender (F/M)	62/130
UPDRS total score (Med-Off)	66.4 (18.5)
UPDRS III (Med-Off)	40.2 (12.0)
UPDRS III (Med-On)	18.8 (9.2)
PIGD (Med-Off)	6.5 (3.6)
UPDRS Item 28 (Med-Off)	1.6 (0.9)
UPDRS Item 28 (Med-On)	1.0 (0.8)

*Values represent mean (SD) or numbers*.

*UPDRS, unified PD rating scale; PIGD, postural instability and gait difficulty score*.

### Testing

The patients were videotaped while performing a walking task following a standardized protocol at baseline (1 month before the implantation of the DBS electrodes) and at 6–24 months after surgery. At baseline, the participants were tested in Med-Off (after 12 h withdrawal of l-dopa and up to 72 h of other anti-Parkinson medication) and Med-On (~60 min after intake of 1.5 times the regular l-dopa dose). At follow-up, testing was performed under Med-Off/Stim-Off, Med-On/Stim-Off, Med-Off/Stim-On, and Med-On/Stim-On conditions. In the Stim-Off conditions, the stimulator had been turned off for ~30 min.

**Figure 1:**
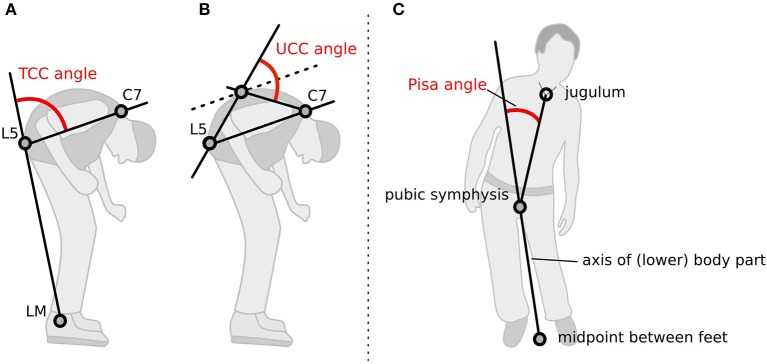
Method how the postural angles were assessed with the NeuroPostureApp: **(A)** total camptocormia (TCC) angle, **(B)** upper camptocormia (UCC) angle, and **(C)** Pisa angle. LM, lateral malleolus.

The participants' postures were video recorded following a standardized protocol. Two screenshots, a lateral and a frontal view, were obtained from the video for each medical treatment condition. A total of 2,304 screenshots were generated, randomly coded, and then used to rate the posture with a free NeuroPostureApp© (http://www.neuroimaging.uni-kiel.de/NeuroPostureApp/) using the following criteria, which are described in detail in an international consensus study regarding the angle measurements for camptocormia (8). The total camptocormia (TCC) angle is defined as the angle between the line connecting the lateral malleolus to the L5 spinous process and the line connecting the L5 spinous process and the C7 spinous process (8). The upper camptocormia (UCC) angle is defined as the angle of the lines between the vertebral fulcrum to the spinous processes of L5 and C7, respectively (8). Lateral deviation (Pisa angle) is defined as the angle between a line between the midpoint of the feet and the pubic symphysis and a line between the pubic symphysis and the jugulum (Figure 1). Two independent raters, blinded to the assessment time point and the test conditions, performed the rating.

PD symptom severity was evaluated with the unified PD rating scale (UPDRS) (10). The postural instability and gait difficulty score (11) was calculated by the sum of the UPDRS items 13, 14, 15, 29, and 30. In addition, an axial subscore was calculated as the sum of the UPDRS items 18, 19, 22, 29, and 30 (12).

The ethics committee of the medical faculty of Kiel University approved the study (reference number D424/18). Informed consent was obtained from all participants.

### Subgroups

For the TCC angle, the following subgroups were considered: TCC-normal, group of patients with a TCC angle within the range of healthy controls [TCC angle < upper 95% confidence interval (95% CI) of healthy controls (10.9°; data from Schlenstedt et al., submitted)]; TCC-stooped, group of patients with a TCC angle above upper 95% CI of healthy control and below 30°; TCC-CC, group of patients with a clinically diagnosed camptocormia (CC) as defined by a TCC angle ≥30° (13).

Similar to the procedure for the TCC subgroups, the following subgroups were defined for the UCC angle: UCC-normal, group of patients with a UCC angle within the range of healthy controls [UCC angle < upper 95% CI of healthy controls (35.4°)]; UCC-stooped, patients with an UCC angle above upper 95% CI of healthy control and below 45°; and UCC-CC, participants with a clinically diagnosis of UCC as defined by an UCC angle ≥45° (14).

For the Pisa angle, we defined: Pisa-normal, group of patients with a Pisa angle within the range of healthy controls [Pisa angle < upper 95% CI of healthy controls (1.2°)]; Pisa-stooped, patients with an Pisa angle above upper 95% CI of healthy control and below 10°; Pisa syndrome, participants with a clinically diagnosis of a Pisa syndrome as defined by a Pisa angle ≥10° (2).

The data for the sex- and age-matched healthy controls (*n* = 78) (Schlenstedt et al., submitted) were obtained in another part of the study project and will be published separately. The angle measurement of the healthy controls was made with the same method as for the PD patients.

### Statistics

To determine the immediate effects of medication and stimulation (study aim I) only the follow-up data containing the four conditions (Med-Off/Stim-Off, Med-On/Stim-Off, Med-Off/Stim-On, and Med-On/Stim-On) were included in the analysis. Linear mixed models were calculated with TCC angle, UCC angle, and Pisa angle as dependent variables, respectively. Medication and stimulation were included as fixed effects and a random intercept for participants. To investigate whether the subgroups improved differently under medication and/or stimulation, a model was ran including the TCC and UCC angles as dependent variables, respectively, and the medication × subgroup and stimulation × subgroup interactions as fixed effects with a random intercept for participants.

To address study aim II, the carryover effect of stimulation from baseline (Med-Off) to follow-up (Med-Off/Stim-Off) was investigated. Again, TCC, UCC, and Pisa angles were included as dependent variables in the linear mixed models, respectively. Time was included as fixed effect with a random intercept for participants. Study aim III investigated the additional effect of stimulation over time (baseline Med-On to follow-up Med-On/Stim-On). TCC, UCC, and Pisa angles were included as dependent variables in the linear mixed models with time as fixed effect and subjects as random intercept, respectively. To investigate whether the effect of medication and stimulation differed between male and female participants, gender was added as additional factor to the linear mixed model used for study aim I, and the gender × medication as well as gender × stimulation interactions were considered. To identify possible predictors of the effect of stimulation, linear regression analyses were conducted with either the difference from baseline Med-Off to follow-up Med-Off/Stim-On or baseline Med-On to follow-up Med-On/Stim-On as dependent variables, respectively. After having checked for multicollinearity, the following independent, predictor variables were included: age, l-dopa responsiveness of UPDRS part III, and l-dopa responsiveness for UPDRS posture item 28. For the linear models, *post-hoc* tests were conducted with Tukey correction for multiple comparisons. The predefined level of significance was set at *p* < 0.05. Statistical analysis was performed with R (version 1.0.136) (15).

## Results

### Spectrum of Postural Alignments in the Examined Patients

Patient characteristics for the entire cohort are shown in Table 1.

For the TCC angle, 11 (5.7%) participants had TCC angles within the range of healthy controls (Schlenstedt et al., submitted) (TCC-normal), 157 (81.8%) patients had a stooped posture (TCC-stooped), and 13 (6.8%) of the 192 participants had a TCC angle ≥30° and were diagnosed as suffering from camptocormia (baseline Med-Off) (13).

For the UCC angle, 37 (19.3%) subjects were considered to be within the range of healthy controls (UCC-normal), 104 (54.2%) had a stooped posture (UCC-stooped), and 40 (20.8%) patients were diagnosed with upper camptocormia defined by an UCC angle ≥45° (baseline Med-Off) (14). Nine (4.7%) participants had both a clinically diagnosed TCC and UCC.

For the Pisa angle, 61 (31.8%) subjects were within the range of healthy controls regarding the lateral bending (Pisa-normal), 120 (62.5%) had a postural alignment between healthy and postural changes, and 2 patients (1.0%) had a Pisa syndrome with an angle of lateral deviation ≥10° (2). Missing values of the postural angles appear due to the inability to stand up and walk independently without any aid in some of the patients or as the quality of some videos was too low to rate them.

### Medication and Stimulation Both Improve Posture

**Figure 2:**
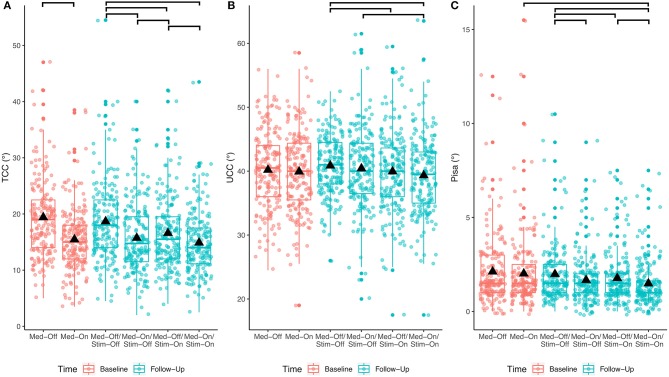
Effects of any treatment condition pre- and post-surgical for the entire PD cohort. The bars at the top show the significant *post-hoc* effects. **(A)** Treatment effects for TCC angle; **(B)** treatment effects for UCC angle; **(C)** treatment effects for Pisa angle.

The results of the immediate effect of medication and stimulation are presented for the follow-up data (study aim I). For the TCC angles post-DBS, a significant effect of medication (*p* < 0.001; *F* = 125.3), a significant effect of stimulation (*p* < 0.001; *F* = 40.3), and a medication × stimulation interaction (*p* = 0.006; *F* = 7.5) were found for the entire cohort (Table 2). Figure 2 shows significant *post-hoc* comparisons. Notably, medication had an effect additional to stimulation (*p* < 0.001).

**Table 2:** Effects of medication and stimulation on the different angles.

**Variable**	**Time**	**Condition**	**All**	**Normal posture^a^**	**Stooped^b^**	**CC^c^**
TCC angle	Baseline	Med-Off	19.4 (7.0)	8.9 (1.6)	18.8 (4.8)	35.9 (5.3)
		Med-On	15.5 (5.6)	7.9 (3.1)	15.3 (4.5)	24.4 (8.2)
	Follow-up	Med-Off/Stim-Off	18.7 (7.2)	11.0 (4.4)	18.5 (6.5)	28.3 (8.0)
		Med-On/Stim-Off	15.7 (6.3)	8.8 (4.6)	15.6 (5.4)	24.7 (8.6)
		Med-Off/Stim-On	16.6 (6.3)	10.5 (3.5)	16.2 (5.4)	25.6 (8.2)
		Med-On/Stim-On	14.9 (5.6)	9.1 (4.1)	14.8 (4.7)	21.0 (9.4)
UCC angle	Baseline	Med-Off	40.2 (6.0)	31.7 (2.6)	40.1 (2.5)	48.4 (2.6)
		Med-On	39.9 (6.2)	34.5 (5.3)	39.5 (5.0)	45.3 (5.1)
	Follow-up	Med-Off/Stim-Off	40.9 (4.6)	39.4 (4.7)	40.8 (4.3)	42.5 (4.6)
		Med-On/Stim-Off	40.4 (6.2)	36.4 (6.7)	39.9 (5.2)	45.1 (5.7)
		Med-Off/Stim-On	39.9 (6.3)	35.8 (6.5)	39.4 (5.3)	44.7 (6.0)
		Med-On/Stim-On	39.3 (6.1)	35.8 (7.2)	38.7 (4.7)	44.0 (6.2)
Pisa angle	Baseline	Med-Off	2.1 (1.9)	NA	NA	NA
		Med-On	1.9 (1.5)	NA	NA	NA
	Follow-up	Med-Off/Stim-Off	2.0 (1.5)	NA	NA	NA
		Med-On/Stim-Off	1.7 (1.3)	NA	NA	NA
		Med-Off/Stim-On	1.8 (1.4)	NA	NA	NA
		Med-On/Stim-On	1.5 (1.2)	NA	NA	NA

*Values represent mean (SD)*.

a *Normal posture: subgroup of patients below the upper 95% CI of healthy controls (TCC angle: n = 11; UCC angle: n = 37)*.

b *Stooped: subgroup of patients with stooped posture (above upper 95% CI of healthy control and for TCC <30° and UCC <45°) (TCC angle: n = 157; UCC angle: n = 104)*.

c *CC: subgroup of patients with clinically diagnosed camptocormia (for TCC: TCC angles ≥30°, n = 13; for UCC: UCC angles ≥45°, n = 14)*.

For the UCC angles, significant effects of medication (*p* = 0.033; *F* = 4.6) and of stimulation (*p* < 0.0001; *F* = 15.5) were found. The medication × stimulation interaction was not significant for UCC (*p* = 0.844; *F* = 0.03). Notably, stimulation had an effect additional to medication (*p* = 0.02).

Regarding the Pisa angle, medication and stimulation both had significant effects (Med: *p* < 0.0001, *F* = 27.2; Stim: *p* < 0.001, *F* = 13.1), whereas there was no significant medication × stimulation interaction (*p* = 0.778, *F* = 0.1). Figure 2 shows that the mean effects of the interventions on the TCC angle are greater than on the UCC and Pisa angles.

### Effects of Interventions Depend on Severity of the Postural Abnormality

**Figure 3:**
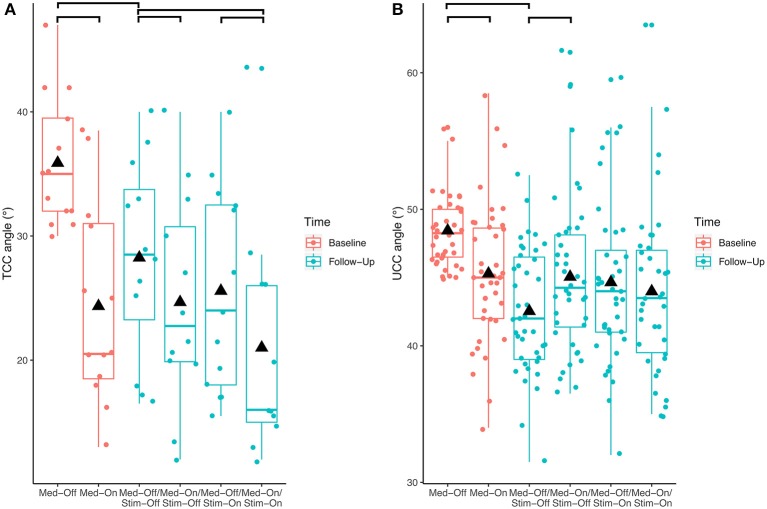
Effects of the different treatment conditions pre- and post-surgical regarding **(A)** total camptocormia (TCC) and **(B)** upper camptocormia (UCC) angles only in those patients with postural disorders (defined for total camptocormia with a TCC angle ≥30° and for upper camptocormia with an UCC angle ≥45°).

Investigating the patients with camptocormia (TCC angles ≥30°) separately showed significant effects of medication (*p* < 0.001, *F* = 17.9) and stimulation (*p* = 0.008, *F* = 8.0) for the TCC angles (Figure 3). Patients with camptocormia were more likely to have a substantial improvement, while those 19% of the patients with normal TCC still remained unchanged. Of the 131 patients with stooped posture, 55 were improved to a normal angle of <10.9° after the intervention. The mean TCC of 35.9° in the 13 patients with camptocormia improved to 21.0°, and two of them improved to within the normal range. When analyzing the patients with upper camptocormia (UCC angles ≥45°) separately, no significant effect of medication or stimulation was found. For the two patients with the Pisa syndrome, no further statistical analysis was performed because of the small group size.

**Figure 4:**
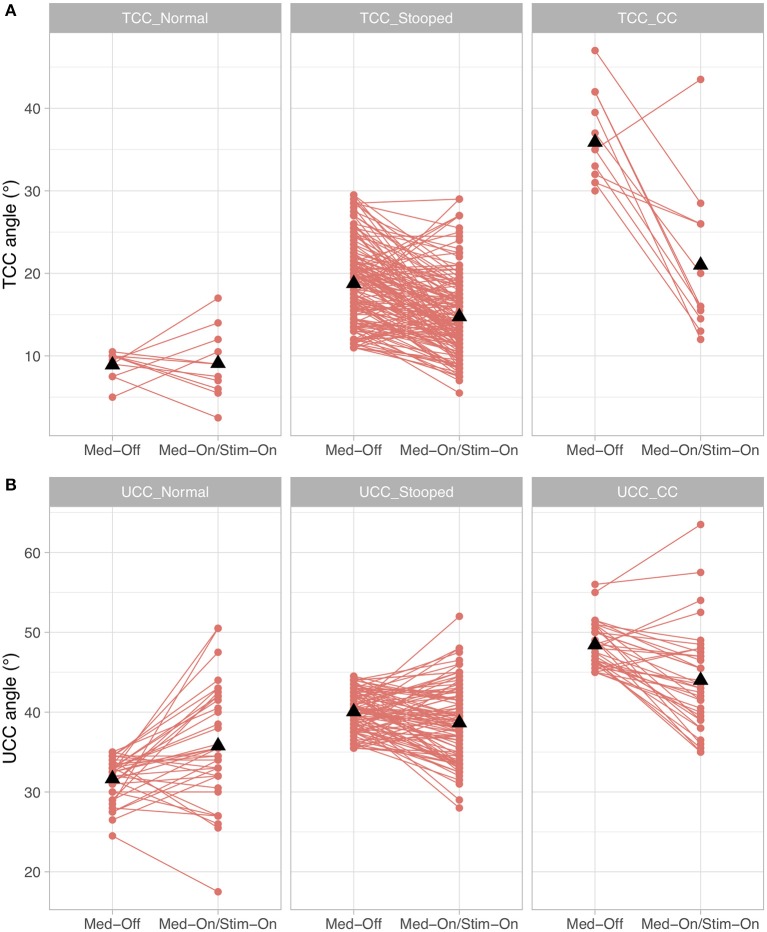
Treatment effects for the conditions presurgical Med-Off and post-surgical Med-On/Stim-On for the three subgroups of Parkinson's disease (PD) patients for **(A)** total camptocormia (TCC) angle and **(B)** upper camptocormia (UCC) angle; TCC_Normal/UCC_Normal: patients with postural alignment within the range of healthy; TCC_Stooped/UCC_Stooped: patients with a stooped posture [above upper 95% confidence interval of healthy and beneath clinical criteria for TCC (30°) or UCC (45°)]; TCC_CC/UCC_CC: patients with clinically diagnosed TCC camptocormia (CC) or UCC CC.

When investigating whether the different subgroups improved differently under medication and stimulation with regard to the TCC angle, a significant medication × subgroup interaction (*p* < 0.0001, *F* = 36.4) and stimulation × subgroup interaction (*p* < 0.0001, *F* = 15.4) were found. For UCC angle, a significant medication × subgroup interaction (*p* < 0.0001, *F* = 13.2) and stimulation × subgroup interaction (*p* < 0.0001, *F* = 11.3) were found. As for the Pisa angle, only *n* = 2 participants had a clinically diagnosed Pisa syndrome, only Pisa-normal and Pisa-stooped were included in the model, revealing a significant medication × subgroup interaction (*p* < 0.0001, *F* = 309.1), but the stimulation × subgroup comparison was not significant (*p* = 0.716, *F* = 0.3). These results indicate that patients with larger TCC or UCC angles improved to a greater extent with both medication and stimulation than patients who were less affected (Figure 4). Furthermore, patients with a lateral deviation in posture had a stronger improvement by medication compared to those with no lateral deviation, but the stimulation effect was similar for the subgroups of Pisa angle.

### Stimulation Has a Carryover Effect for TCC Angle in Patients With Camptocormia

With respect to the carryover effect from baseline (Med-Off) to follow-up (Med-Off/Stim-Off) (study aim II), no significant effects of time were found for TCC (*p* = 0.109, *F* = 2.6), UCC (*p* = 0.153, *F* = 2.1), or Pisa angles (*p* = 0.969, *F* = 0.0) when the entire sample was analyzed. However, a significant carryover effect was found (*p* < 0.01, *F* = 9.5) when the patients with camptocormia (TCC angles ≥30°) were analyzed separately (Figure 3).

### DBS Has an Effect on Pisa Syndrome Over Time Additional to Medication

With respect to the comparison from baseline Med-On to follow-up Med-On/Stim-On (study aim III), a significant effect of time was found for the Pisa angle (*p* < 0.0001, *F* = 21.6) but not for the TCC (*p* = 0.125, *F* = 2.4) or UCC angles (*p* = 0.176, *F* = 1.8) when all participants were included in the analysis.

### Female and Male Participants Benefit Similarly by Medication and Stimulation

There was no significant gender × stimulation interaction for the TCC angle (*p* = 0.449 *F* = 0.6), indicating that male and female patients benefit similarly from DBS. Although a significant interaction was found for gender × medication (*p* = 0.047, *F* = 3.1), the *post-hoc* comparison revealed no significant differences between male and female when comparing the changes from Med-Off to Med-On. With regard to the UCC and Pisa angles, there were no significant gender × medication or gender × stimulation interactions.

### Predictors of the Effect of Stimulation

We included age and responsiveness of UPDRS III and UPDRS Posture Item 28 to l-dopa as independent variables in a linear regression model with the DBS effect on TCC, UCC, or Pisa angles as dependent variables, respectively. For TCC, the model was significant (*p* = 0.006), but predictability was low (*R*^2^ = 0.05). The models were not significant for UCC (*p* = 0.09) or Pisa angles (*p* = 0.09).

## Discussion

We examined a large cohort of PD patients with advanced disease using the video and photo material taken during standardized l-dopa trials before and after DBS in the subthalamic nucleus. The patients were characterized with regard to two important postural alignments: the forward bending differentiated in the TCC and the UCC as well as the lateral bending referred to as the “Pisa angle.” These terms were taken from an international consensus paper (8) and indicate here the defined angles; they should not be confused with the postural disorders themselves.

### Effects of Medication and DBS on Postural Alignments for the Entire Cohort of PD Patients

With respect to the immediate effects of medication and stimulation, we found statistically significant effects for the TCC angles in the entire cohort when comparing the Med-Off/Stim-Off condition with every other interventional condition at follow-up. The effect of Med-On/Stim-On was better than the effect of the stimulation alone (Med-Off/Stim-On). On average, the overall effects are very limited. Roediger et al. (7) used a different method for assessing the angle but came up with rather similar conclusions. The authors described an improvement of 6.7% between the pre- and postsurgical assessments. Other studies (16, 17) used item 28 (posture) of the UPDRS III as the main outcome parameter and included patients with a score >0 or ≥2, respectively. These results cannot be compared with studies evaluating the angles qualitatively with scores as they only roughly correlate with angle measurements (Schlenstedt et al., submitted).

For the UCC angle, we found no significant effect for the comparison of the Med-Off/Stim-Off and Med-On/Stim-Off conditions. The effect of DBS alone and the combined therapy with medication and stimulation did not differ statistically.

For the Pisa angle, the results of the comparison of the various treatment conditions very closely resembled the findings for the TCC angle.

Interestingly, an additional effect of stimulation beyond that of the medication was found for the Pisa angle, but not for the other two postural angles. This may indicate a stronger effect of DBS on postural alignment in the mediolateral rather than the anteroposterior direction.

We did not find any gender differences for the treatment effects, indicating that women and men both improved similarly with medication and stimulation.

The predictability of the effect of stimulation on the TCC was low when considering the baseline l-dopa responsiveness of the TCC as the outcome parameter. A different study suggested l-dopa responsiveness, disease duration, and gender as possible predictors of the effect of stimulation (7). However, also in that study, only 37% of the variance of the stimulation effect was predicted (7). A study with a great number of PD patients found age, duration of disease, H-Y stage, pain, and vertebral disease relevant, but without further specification of their individual relevance (18).

### Treatment Effects in Subgroups of Patients With Postural Disorders

These results need to be interpreted clinically. The first question is to understand the value of treatment with regard to the different angles measured in this study. We have shown in a previous paper (13) that patients with PD without camptocormia have a TCC angle of <30°, and this was used here as the objective criterion—patients above this angle suffer from camptocormia. On the other hand, normal bending and bending in PD as stooped posture was separated as the 95% confidence limit of the normal population (Schlenstedt et al., submitted), which was 10.9°. According to these definitions, 6.8% of our cohort were camptocormia patients (13). This prevalence fits the reported prevalence rates described in the literature (19).

For forward bending, the upper 95% confidence limits of the normal UCC and Pisa angle were found to be 35.4 and 2°, respectively. Unfortunately, data-based cutoff criteria for the definition of the upper camptocormia and Pisa syndrome are lacking for the UCC and the Pisa angle, respectively. Instead, we still have to base them on expert rating with proposed ≥45° for the UCC (14) and ≥10° for the Pisa angle (2). According to these cutoff criteria, 7.3% of our PD patient cohort suffer from upper camptocormia and 1.0% patients from Pisa syndrome. While some studies (20) report a much higher prevalence for the Pisa syndrome, another current study described the same rate as ours (7).

The main findings of our study are that (1) the two interventions and their combination provided only minor improvements of the three angles in patients with normal posture, (2) a significant improvement for the three angles was seen in patients with abnormal posture (TCC angles between 10.9 and 29%), but the extent of these changes was also small with 4° on average, and (3) the most profound changes were found in the small group of 13 patients with camptocormia (TCC angle ≥30°) with a significant average TCC improvement of 14.9° from baseline Med-Off to follow-up Med-On/Stim-On. Furthermore, for the TCC angle, the effects of the combined intervention were slightly larger than the stimulation effect alone.

Treatment effects have been studied in several cohorts with Parkinsonian camptocormia patients. Small studies have been published with varying clinical or quantitative measurement methods confirming an effect of l-dopa (3, 12, 21). On the other hand, there is also a body of literature arguing against the positive effect of l-dopa in the camptocormia of PD (22, 23). The reason for discrepancies is difficult to comment on as long as we have no uniform measurement methods. Likewise, open studies have found a significant improvement for camptocormia with DBS, but again, the angle measurements were based on varying measurement methods (24–26). A comparable measure was used by one study (7), and for their three patients with camptocormia, they found improvements similar to those in our cohort.

Concerning the patients with an UCC angle ≥45°, we were unable to detect any significant beneficial effects for any of the treatment conditions. In fact, the UCC angle increased from Med-Off/Stim-Off and Med-On/Stim-Off. Roediger et al. (7) reported an improvement in the ventral thoracic angle (comparable to the UCC), but this was based on only two patients in their cohort and was without statistical significance.

Owing to the small number of Pisa patients in our cohort, we did not conduct any further statistical analyses regarding the Pisa syndrome.

To summarize the treatment effects on postural disorders, we only found strong treatment effects in camptocormia patients with a TCC angle ≥30°, which emphasizes a positive influence of medication and stimulation on posture in the more severely affected patients. In addition, in contrast to the entire cohort, we found a carryover effect in the group of patients with camptocormia (TCC angle ≥30°). We interpret this finding as an indication that treatment with DBS affects posture in this particular subgroup over time.

### Impact of This Study on Criteria to Classify Postural Impairments

This study used three different angles (TCC, UCC, and the Pisa angle) to capture the postural abnormalities of PD patients. The treatment effects were most clearly demonstrated for the TCC angle. Figure 3 shows that the improvements of this angle in patients with a stooped posture were frequently great enough as to regain a normal posture. Interestingly, these patients did not show a TCC angle larger than 30° under any treatment condition. This was found to delineate between the stooped posture of PD and camptocormia in a different cohort (13) and was empirically confirmed here. It is a possibly an indication that a different pathological process is involved in camptocormia in addition to the stooped posture of PD. On the other hand, patients with camptocormia may still improve substantially even to regaining a normal posture under the combined intervention nurturing hopes for therapeutic success for this devastating symptom.

For the lateral deviation, we used the so-called Pisa angle and found only small changes under treatment. In our patient cohort with advanced PD, only two patients had a clear-cut Pisa syndrome, and conclusions are impossible with so few patients. Larger patient groups need to be assessed with these measures.

### Limitations

Despite the very large number of subjects and patients with PD, the chosen population may not represent the full spectrum of the disease as all the patients had been selected for DBS. Larger groups of patients without this selection criterion need to be assessed. We used representative pictures of our screen shot from the patient videos, and these are certainly prone to subjectivity. However, they were taken by experienced clinicians, who carefully made certain that they were representative. The different angles are measures from anatomical landmarks, which are not always easy to spot. However, the high reliability between the two raters indicates that this is an only minor limitation. X-rays as the gold standard for trunk bending are not appropriate as routine clinical measures.

## Conclusions

Detailed quantitative angle measurements with the free NeuroPostureApp (http://www.neuroimaging.uni-kiel.de/NeuroPostureApp/) are sensitive to treatment effects in PD. Both medication and stimulation improved postural alignment in anteroposterior and mediolateral direction in PD. In particular, treatment effects were strongest for postural impaired patients, and for a large portion, this led to a normal posture. The effects were even stronger for patients with camptocormia. Female and male patients both improve similarly by the two treatments. The TCC angle is a valid measure of the postural abnormalities underlining the objective separation between stooped posture of PD and camptocormia and was shown here to be sensitive to change. The UCC angle was less sensitive but may be a useful assessment tool for patients with isolated upper camptocormia. The Pisa angle was measured, but further investigation is needed to better understand the mediolateral abnormalities in PD.

## Data Availability Statement

The datasets generated for this study will not be made publicly available and will be used in a further project that is not published right now.

## Ethics Statement

The studies involving human participants were reviewed and approved by ethics committee of the Medical Faculty of Kiel University. The patients/participants provided their written informed consent to participate in this study.

## Author Contributions

CS: statistics, analyzing of the data, and writing. OGa: data collection and writing. KB: data collection and statistics. RW: data collection and critical review of the first draft. OGr: development of the NeuroPostureApp. GD and NM: study design, analyzing of the data, and writing.

### Conflict of Interest

The authors declare that the research was conducted in the absence of any commercial or financial relationships that could be construed as a potential conflict of interest.

## References

[1] Benatru I Vaugoyeau M Azulay JP. Postural disorders in Parkinson's disease. Neurophysiol Clin. (2008) 38:459–65. 10.1016/j.neucli.2008.07.006 19026965

[2] Doherty KM van de Warrenburg BP Peralta MC Silveira-Moriyama L Azulay JP Gershanik OS. Postural deformities in Parkinson's disease. Lancet Neurol. (2011) 10:538–49. 10.1016/S1474-4422(11)70067-9 21514890

[3] Kataoka H Ueno S. Can postural abnormality really respond to levodopa in Parkinson's disease? J Neurol Sci. (2017) 377:179–84. 10.1016/j.jns.2017.04.025 28477691

[4] Curtze C Nutt JG Carlson-Kuhta P Mancini M Horak FB. Levodopa is a double-edged sword for balance and gait in people with Parkinson's disease. Mov Disord. (2015) 30:1361–70. 10.1002/mds.26269 26095928 PMC4755510

[5] Nantel J McDonald JC Bronte-Stewart H. Effect of medication and STN-DBS on postural control in subjects with Parkinson's disease. Parkinsonism Relat Disord. (2012) 18:285–9. 10.1016/j.parkreldis.2011.11.005 22130147

[6] Visser JE Allum JH Carpenter MG Esselink RA Speelman JD Borm GF. Subthalamic nucleus stimulation and levodopa-resistant postural instability in Parkinson's disease. J Neurol. (2008) 255:205–10. 10.1007/s00415-008-0636-x 18274810

[7] Roediger J Artusi CA Romagnolo A Boyne P Zibetti M Lopiano L. Effect of subthalamic deep brain stimulation on posture in Parkinson's disease: a blind computerized analysis. Parkinsonism Relat Disord. (2019) 62:122–7. 10.1016/j.parkreldis.2019.01.003 30638820

[8] Margraf NG Wolke R Granert O Berardelli A Bloem BR Djaldetti R. Consensus for the measurement of the camptocormia angle in the standing patient. Parkinsonism Relat Disord. (2018) 52:1–5. 10.1016/j.parkreldis.2018.06.013 29907329

[9] Hughes AJ Daniel SE Kilford L Lees AJ. Accuracy of clinical diagnosis of idiopathic Parkinson's disease: a clinico-pathological study of 100 cases. J Neurol Neurosurg Psychiatry. (1992) 55:181–4. 10.1136/jnnp.55.3.181 1564476 PMC1014720

[10] Fahn S Elton RL Unified Parkinson's disease rating scale In: Fahn S Marsden CD Calne D Goldstein M, editors. Recent Developments in Parkinson's Disease. Florham Park, NJ: MacMillan Health Care Information (1987). p. 153–63.

[11] Jankovic J McDermott M Carter J Gauthier S Goetz C Golbe L. Variable expression of Parkinson's disease: a base-line analysis of the DATATOP cohort. The Parkinson Study Group. Neurology. (1990) 40:1529–34. 10.1212/WNL.40.10.1529 2215943

[12] Bloch F Houeto JL Tezenas du Montcel S Bonneville F Etchepare F Welter ML. Parkinson's disease with camptocormia. J Neurol Neurosurg Psychiatry. (2006) 77:1223–8. 10.1136/jnnp.2006.087908 16754693 PMC2077378

[13] Margraf NG Granert O Hampel J Wrede A Schulz-Schaeffer WJ Deuschl G. Clinical definition of camptocormia in Parkinson's disease. Mov Disord Clin Pract. (2017) 4:349–57. 10.1002/mdc3.12437 30363363 PMC6174367

[14] Fasano A Geroin C Berardelli A Bloem BR Espay AJ Hallett M. Diagnostic criteria for camptocormia in Parkinson's disease: a consensus-based proposal. Parkinsonism Relat Disord. (2018) 53:53–7. 10.1016/j.parkreldis.2018.04.033 29759930 PMC7293065

[15] R Development Core Team. R: A Language and Environment for Statistical Computing. Vienna: R Foundation for Statistical Computing (2010).

[16] Artusi CA Zibetti M Romagnolo A Rizzone MG Merola A Lopiano L. Subthalamic deep brain stimulation and trunk posture in Parkinson's disease. Acta Neurol Scand. (2018) 137:481–7. 10.1111/ane.12889 29285760

[17] Umemura A Oka Y Ohkita K Yamawaki T Yamada K. Effect of subthalamic deep brain stimulation on postural abnormality in Parkinson disease. J Neurosurg. (2010) 112:1283–8. 10.3171/2009.10.JNS09917 19895200

[18] Ando Y Fujimoto KI Ikeda K Utsumi H Okuma Y Oka H. Postural abnormality in Parkinson's disease: a large comparative study with general population. Mov Disord Clin Pract. (2019) 6:213–21. 10.1002/mdc3.12723 30949552 PMC6417750

[19] Srivanitchapoom P Hallett M. Camptocormia in Parkinson's disease: definition, epidemiology, pathogenesis and treatment modalities. J Neurol Neurosurg Psychiatry. (2016) 87:75–85. 10.1136/jnnp-2014-310049 25896683 PMC5582594

[20] Tinazzi M Fasano A Geroin C Morgante F Ceravolo R Rossi S Italian Pisa Syndrome Study Group. Pisa syndrome in Parkinson disease: an observational multicenter Italian study. Neurology. (2015) 85:1769–79. 10.1212/WNL.0000000000002122 26491088

[21] Djaldetti R Mosberg-Galili R Sroka H Merims D Melamed E. Camptocormia (bent spine) in patients with Parkinson's disease–characterization and possible pathogenesis of an unusual phenomenon. Mov Disord. (1999) 14:443–7. 10.1002/1531-8257(199905)14:3<443::AID-MDS1009>3.0.CO;2-G 10348467

[22] Seki M Takahashi K Koto A Mihara B Morita Y Isozumi K. Parkinson's disease, camptocormia in Japanese patients with Parkinson's disease: a multicenter study. Mov Disord. (2011) 26:2567–71. 10.1002/mds.23955 21953897

[23] Azher SN Jankovic J. Camptocormia: pathogenesis, classification, and response to therapy. Neurology. (2005) 65:355–9. 10.1212/01.wnl.0000171857.09079.9f 16087897

[24] Sako W Nishio M Maruo T Shimazu H Matsuzaki K Tamura T. Subthalamic nucleus deep brain stimulation for camptocormia associated with Parkinson's disease. Mov Disord. (2009) 24:1076–9. 10.1002/mds.22529 19353719

[25] Asahi T Taguchi Y Hayashi N Hamada H Dougu N Takashima S. Bilateral subthalamic deep brain stimulation for camptocormia associated with Parkinson's disease. Stereotact Funct Neurosurg. (2011) 89:173–7. 10.1159/000324907 21494070

[26] Capelle HH Schrader C Blahak C Fogel W Kinfe TM Baezner H. Deep brain stimulation for camptocormia in dystonia and Parkinson's disease. J Neurol. (2011) 258:96–103. 10.1007/s00415-010-5695-0 20803027

